# Modified Qianghuo Shengshi Decoction Ameliorates Osteoarthritis via Inhibiting PI3K/Akt Pathway‐Related Ferroptosis

**DOI:** 10.1111/jcmm.70691

**Published:** 2025-06-30

**Authors:** Chen Zhuang, Enli Li, Wen‐kai Li, Xiaojuan Geng, Chenxuan Hong, Yu Pan, Lei Yang

**Affiliations:** ^1^ Alberta Institute Wenzhou Medical University Zhejiang China; ^2^ Department of Orthopaedics The Second Affiliated Hospital and Yuying Children's Hospital of Wenzhou Medical University Wenzhou China; ^3^ The First School of Clinical Medicine Zhejiang Chinese Medical University Zhejiang Hangzhou China; ^4^ School of Pharmacy Zhejiang Chinese Medical University Zhejiang Hangzhou China; ^5^ Department of Orthopaedics Cangnan Hospital Affiliated to Wenzhou Medical University Zhejiang China; ^6^ Quzhou TCM Hospital, Junction of Four Provinces Affiliated to Zhejiang Chinese Medical University Zhejiang China; ^7^ Quzhou Hospital of Traditional Chinese Medicine Zhejiang China; ^8^ Department of Orthopaedics The First Affiliated Hospital of Zhejiang Chinese Medical University, Zhejiang Provincial Hospital of Traditional Chinese Medicine Zhejiang Hangzhou China

**Keywords:** ferroptosis, modified qianghuo shengshi decoction, osteoarthritis, PI3K/Akt, TCM

## Abstract

Modified Qianghuo Shengshi decoction (MQSD), a TCM formula, is clinically used for osteoarthritis (OA) symptom relief. Its exact molecular actions, however, are not fully understood. This investigation seeks to elucidate the molecular mechanisms by which MQSD impedes OA progression. High performance liquid chromatography (HPLC) was employed to delineate MQSD's chemical profile. The CCK‐8 assay determined MQSD's impact on chondrocyte viability in the presence or absence of IL‐1β. EDU and Annexin V‐FITC assays evaluated chondrocyte proliferation and apoptosis, respectively. Alcian Blue staining probed extracellular matrix (ECM) secretion by chondrocytes. Network pharmacology was utilised to pinpoint disease targets, while various assays assessed ferroptosis‐related chondrocyte phenotypes under Erastin or Ferrostatin‐1 treatments. IHC, qRT‐PCR and western blot analyses were conducted to ascertain MQSD's cellular effects. Micro‐CT and Safranin O Fast Green staining provided insights into knee joint morphology and cartilage integrity. Molecular docking assessed the binding affinity of MQSD's active compounds to PI3K/Akt/GPX4. MQSD promotes chondrocyte proliferation, prevents apoptosis, and enhances viability. It further modulates the ECM output and the anabolic‐to‐catabolic ratio within chondrocytes. Through network pharmacology, ferroptosis linked to the PI3K/Akt pathway is identified as crucial. Pre‐application of MQSD elevates the GSH/GSSG ratio and mitochondrial density, while it reduces Fe^2+^ levels, ROS, and lipid droplet accumulation in chondrocytes. Moreover, MQSD activates GPX4 and inhibits p‐AKT levels. Molecular docking studies affirm the strong interaction between MQSD's key ingredients and the PI3K/Akt/GPX4 pathway components. Through modulation of ferroptosis via the PI3K/Akt pathway, MQSD retards OA progression.

**Abbreviations**
BP: biological processes
BV/TV: bone volume fraction
CC: cellular components
Col2: type II collagen
CoQ10: coenzyme Q10
DFO: deferoxamine
DRG: dorsal root ganglion
ECM: extracellular matrix
FBS: fetal bovine serum
Fer‐1: Ferrostatin‐1
FSP‐1: ferroptosis suppressor protein
GO: gene ontology
GPX4: Glutathione peroxidase 4
HPLC: high performance liquid chromatography
IHC: immunohistochemical
IL‐1: interleukin‐1
KEGG: Kyoto encyclopedia of genes and genomes
MDA: malondialdehyde.
MF: molecular functions
MMP13: matrix metalloproteinase13
MMP‐3: matrix metalloproteinase‐3
MQSD: modified qianghuo shengshi decoction
NSAIDs: nonsteroidal antiinflammatory drugs
OA: osteoarthritis
OARSI: Osteoarthritis Research Society International
PDB: Protein Data Bank
RA: rheumatoid arthritis
ROS: reactive oxygen species
SFs: synovial fibroblasts
Tb.N: bone trabeculae
Tb.Th: bone trabecular thickness
TCM: traditional Chinese medicine
TNF‐α: tumourtumor necrosis factor‐α

## 1 Introduction

Osteoarthritis (OA) constitutes a severe joint disorder marked by the progressive degeneration of cartilage tissue, as highlighted by Kloppenburg [1]. This condition arises from age‐associated alterations, traumatic damages, and the demise of chondrocytes due to a spectrum of physical and chemical provocations, culminating in the disruption of cartilage equilibrium and accelerating OA's progression [2, 3]. Globally, the prevalence of OA afflicts over 500 million adults, with China reporting a significant 61.2 million cases [4]. The current scarcity of specific therapeutic strategies for OA has catalysed growing interest in exploring Traditional Chinese Medicine (TCM) as an adjunctive treatment modality [5]. Noteworthy is the accumulating body of evidence underscoring the potential of various Chinese herbal concoctions in sustaining chondrocyte viability and mitigating the progression of OA [6, 7, 8, 9]. This situation underscores the imperative need for a rigorous examination and validation of TCM‐based interventions. By harnessing clinical insights, the aim is to elucidate treatments that exhibit substantial efficacy while minimising adverse reactions, thus enhancing the arsenal of therapeutic strategies for OA management, in concordance with the rigorous scholarly criteria of Phytomedicine.

TCM formulations, primarily consisting of phyto‐derived components, have shown significant therapeutic potential in OA management, notably Modified Qianghuo Shengshi Decoction (MQSD). Esteemed for its historical use in China, MQSD addresses various pain‐associated conditions, including OA, leveraging its anti‐inflammatory and analgesic effects. Empirical research has highlighted MQSD's efficacy, especially in combination with acupressure, leading to marked improvements in pain metrics, joint functionality, and decreases in interleukin‐1 (IL‐1), tumour necrosis factor‐alpha (TNF‐α), and matrix metalloproteinase‐3 (MMP‐3) concentrations in the joint fluid of OA sufferers [10]. Further, preclinical models suggest MQSD's effects might be orchestrated via the modulation of the MAPKs/CREB pathway in the dorsal root ganglion (DRG) and spinal cord [11]. The key botanical ingredient in MQSD, Notopterygium incisum (Qianghuo), has been identified for its pronounced anti‐inflammatory, analgesic, and antioxidant capabilities [12, 13], potentially through the mitochondrial modulation of NLRP3 inflammasome activation [14]. These findings strongly advocate for further investigation into MQSD as a viable OA therapy.

Emerging evidence highlights iron‐dependent programmed cell death, known as ferroptosis, as a pathological process within OA cartilage [15, 16]. Glutathione peroxidase 4 (GPX4) plays a pivotal role in OA pathology, being indispensable for the neutralisation of reactive oxygen species (ROS). Notably, GPX4 concentration is directly linked to enhanced cellular resilience against oxidative stress [17, 18]. Suppression of GPX4 within cartilage leads to exacerbation of extracellular matrix (ECM) degradation through the MAPK/NF‐κB signalling pathway [16]. Moreover, ferroptosis in chondrocytes is associated with an upregulation of matrix metalloproteinase‐13 (MMP13) and a reduction in type II collagen (Col2) levels. Ferrostatin‐1 (Fer‐1), a well‐known ferroptosis inhibitor, mitigates IL‐1β‐driven downregulation of Col2 and upregulation of MMP13 [19]. Furthermore, intra‐articular administration of deferoxamine (DFO), an iron‐binding agent, in OA mouse models promotes Col2 synthesis and prevents chondrocyte apoptosis and cartilage deterioration triggered by erastin, a ferroptosis inducer [20]. Additionally, in mice with conditional GPX4 knockout, injections into the joint space of ferroptosis suppressor protein 1 (FSP‐1) along with coenzyme Q10 (CoQ10) significantly retard OA progression [19].

The PI3K/AKT signalling pathway plays a dual role in the pathogenesis of OA; it promotes chondrocyte proliferation and inhibits apoptosis, thus mitigating OA symptoms. Conversely, pathway inhibition facilitates the restoration of cartilage equilibrium, diminishes subchondral bone osteosclerosis, and enhances autophagy and anti‐inflammatory responses [21, 22]. Downstream, the mTOR and NF‐κB pathways respectively govern autophagy initiation and inflammatory responses in OA [23, 24]. In oncology, two studies have highlighted that downstream effects mediated by PI3K/AKT, specifically through the SREBP1/SCD1 and Rag‐mTORC1‐4EBP pathways, deter ferroptosis. Notably, the latter pathway fosters GPX4 expression [25, 26]. Intriguingly, research in rheumatoid arthritis (RA) has revealed that Semaphorin 5A in RA synovial fluid curtails ferroptosis in RA synovial fibroblasts by tweaking the PI3K/AKT/mTOR axis to boost GPX4 levels [27]. These insights necessitate further explorations to discern the involvement and regulation of ferroptosis by the PI3K/AKT/GPX4 axis in the context of MQSD's effect on delaying OA progression.

Consequently, the foremost objective of this study is to validate the therapeutic potential of MQSD in the management of OA and to elucidate its mechanistic underpinnings via extensive biosignature profiling and molecular docking methodologies, further bolstered by stringent in vivo and in vitro evaluations. This research aspires to integrate innovative, empirically verified TCM approaches, rooted in phyto‐derived substances, into the repertoire of treatment options for OA. In doing so, it aims to augment and refine the array of therapeutic modalities for combating this incapacitating ailment.

## 2 Materials and Methods

### 2.1 Reagents

Fetal bovine serum (FBS, Sa211.02) and DMEM/F12 (CGM104.05), culture medium were sourced from CellMax (Beijing, China). IL‐1β (HY‐P70586), Ferrostatin‐1 (HY‐100579), Erastin (HY‐15763), LY294002 (HY‐10108) and Recilisib (HY‐101625) were obtained from MedChemexpress (USA). Primary antibodies targeting Col2 (ab34712), MMP13 (ab39012), and GPX4 (ab125066) were acquired from Abcam (UK), while AKT (10176‐2‐AP) and p‐AKT (28731‐1‐AP) antibodies were purchased from Proteintech (Wuhan, China). The Cell Counting Kit‐8 was procured from Bioss (BA00208, Beijing, China), and the EdU‐647 Cell Proliferation Assay Kit was obtained from Epizyme Biotech (CX004, Shanghai, China). The MDA Assay Kit was sourced from Nanjing Jiancheng Bioengineering Institute (A003‐1, Nanjing, China), and the Iron Colorimetric Assay Kit was obtained from Applygen Technologies (E1042, Beijing, China). MitoTracker Red CMXRos (C1049B), Annexin V‐FITC Apoptosis Detection Kit (C1062L), ROS Assay Kit (S0033S) and GSH/GSSG Assay Kit (S0053) were purchased from Beyotime Biotechnology (Shanghai, China). Modified Saffron‐O and Fast Green Stain Kit (For Bone) (G1371), Alcian Blue Stain Kit (G1560) and Lipid Fluorescent Staining Kit (Nile Red Method) (G1264) were obtained from Solarbio (Beijing, China).

### 2.2 Cell Extraction and Culture Procedure

Knee cartilage harvested from C57BL/6 mice was dissected into fragments measuring 0.2–0.5 mm. The cartilage pieces underwent a 20‐min digestion in 0.25% trypsin, followed by a wash in antibiotic‐containing PBS. A further digestion step involved treating these fragments with 0.2% type II collagenase in Dulbecco's Modified Eagle Medium (DMEM) for around 40 min at 37°C. Digestion was ceased upon detecting free cells under an inverted microscope, through the addition of FBS. Subsequent dissociation of the cellular mass into single cells was achieved by filtration, collecting the filtrate. This filtrate underwent centrifugation at 1000 rpm for 5 min, discarding the supernatant thereafter. A repeat centrifugation step under the same conditions was performed to obtain primary chondrocytes, which were then propagated in a DMEM/F12 medium enriched with 10% FBS and 1% antibiotics.

### 2.3 Assessment of Cell Viability

Chondrocytes were plated in 96‐well plates at a concentration of 1 × 10^4^ cells per well, setting up six replicates for every experimental setup. After finalising the cell treatments, a viability assay was performed in accordance with the Cell Counting Kit‐8 (CCK‐8) kit's manufacturer guidelines. The assessment of cell viability was based on absorbance measurements at 450 nm using a plate reader.

### 2.4 EdU‐647 Assay for Cell Proliferation

Cell samples were prepared and treated with EdU working solution, followed by an incubation period of 3 h. Subsequently, the cells were fixed, washed, and permeabilized. Click reaction solution was added and the cells were incubated for 30 min at room temperature, shielded from light. Afterward, the cells were washed three times to remove excess reagents. Hoechst 33342 staining solution was then added and the cells were incubated at room temperature for 10 min. Following incubation, the Hoechst 33342 solution was aspirated and the cells were washed three times with washing solution in preparation for fluorescence detection.

### 2.5 Alcian Blue Staining

Cells were fixed in paraformaldehyde for 15 min. Subsequently, the cells were washed three times with PBS. The cells were then immersed in Alcian acidified solution for 3 min, followed by staining with Alcian Blue staining solution for 30 min. After staining, the cells were rinsed six times with PBS. The optical density of the extracted dye was measured at 620 nm.

### 2.6 Annexin V‐FITC/PI Assay for the Apoptosis Rate

Cell samples were prepared, ensuring the preservation of the treated cell culture. Following trypsin digestion, the retained cell culture fluid was added to halt the digestion process. Subsequently, the cells were centrifuged, and the supernatant was discarded, while the cells were collected. The cells were then resuspended in Annexin V‐FITC binding solution, followed by the sequential addition of Annexin V‐FITC and PI staining solution. The cells were incubated for 20 min in darkness, and the results were analysed using flow cytometry.

### 2.7 Nile Red Staining

Cell samples were prepared and fixed appropriately. Following fixation, the cells were treated with an adequate amount of Nile Red staining solution and incubated at room temperature while avoiding exposure to light for 10 min. Subsequently, the cells were washed three times with PBS. Hoechst 33342 staining solution was then added, and the cells were further incubated for 10 min followed by another round of PBS washing (three times). Finally, the stained cells were observed, recorded and imaged under a fluorescence microscope.

### 2.8 GSH and GSSG Assay

Fresh cell samples were prepared for analysis. Using a 96‐well plate, the samples or standards were sequentially added and incubated with glutathione working solution. The absorbance was measured at 412 nm using a spectrophotometer. A standard curve was generated, and the content of total GSH and GSSG was calculated accordingly.

### 2.9 MDA Assay

Cell samples were prepared and subjected to treatment with thiobarbituric acid at 95°C for 80 min. Subsequently, the absorbance at 532 nm was measured using a spectrophotometer. The concentration of MDA was calculated based on the absorbance readings obtained.

### 2.10 Fe^2+/3+^ Analysis

Cell samples were prepared by adding iron colorimetric working solution to each sample, followed by the addition of 30 μL of iron ion detector. The mixture was thoroughly mixed and then incubated at room temperature. Subsequently, the absorbance was measured at 550 nm using a spectrophotometer. A standard curve was plotted using known concentrations of iron ions, and the concentration of iron ions in the samples was calculated accordingly.

### 2.11 ROS Assay

Cell samples were prepared by adding the appropriate volume of 10 μmol/L DCFH‐DA to each sample. The cells were then incubated at 37°C for 20 min and washed three times with DMEM/F12. Subsequently, Hoechst 33342 live cell staining solution was added, and the cells were incubated for an additional 10 min before being washed with PBS three times. Fluorescence microscopy was utilised for observation and imaging.

### 2.12 MitoTracker Assay

Cell samples were prepared and treated with MitoTracker Red CMXRos Working Solution, followed by incubation at 37°C for 30 min. Subsequently, the working solution was removed, and the cells were treated with Hoechst 33342 staining solution and further incubated for 10 min. After incubation, the cells were washed three times with PBS and supplemented with pre‐warmed DMEM/F12 at 37°C. Fluorescence microscopy was employed for observation and image capture of the stained cells.

### 2.13 Preparation and Quality Control of MQSD and Its Drug‐Containing Serums

MQSD comprises eight traditional Chinese herbs as listed in Table 1, with botanical nomenclature authenticated via the World Flora Online database (http://www.worldfloraonline.org, accessed April 1, 2024). All raw materials were obtained from the pharmacy of the First Affiliated Hospital of Zhejiang Chinese Medical University (Hangzhou, China). The herbs were mixed in prescribed ratios, soaked in four times their volume of purified water for 30 min and decocted twice (30 min each time). The combined filtrates were concentrated to 100 mL at 55°C using rotary evaporation and then lyophilized in a freeze‐dryer. The resulting dried extract was stored at −80°C for further use.

**TABLE 1:** The list of herbal names in MQSD.

Chinese name	Botanical name (the plant list)	Part used	Weight (g)
Qianghuo	Notopterygium incisum Ting ex H.T.Chang	Root	15
Fangfeng	Saposhnikovia divaricate (Trucz.) Schischk	Root	10
Duhuo	Heracleum hemsleyanum Diels	Root	15
Chuanxiong	Ligusticum sinense ‘Chuanxiong’	Root	10
Cangzhu	Atractylodes lancea DC	Rhizome	10
Gancao	Glycyrrhiza uralensis Fisch. ex DC	Root	3
Manjinzi	*Vitex rotundifolia*	Seed	6
Gaoben	Ligusticum sinense Oliv	Rhizome	6

**FIGURE 1:**
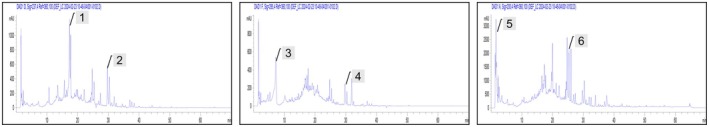
Representative chromatogram of major compounds in MQSD. (1) Cimicifugoside; (2) Compound Glycyrrhizin; (3) 5‐Hydroxymethylfurfural; (4) Osthole; (5) Notopterol; (6) Oxypeucedan hydrate.

Preparation of MQSD‐containing serum: Twenty male SPF‐grade Sprague–Dawley (SD) rats (3–7 weeks old, 50–250 g, purchased from Shanghai BK Laboratory Animal Co. Ltd.) were randomly divided into two groups: the MQSD group and the saline group, with 10 rats in each. Rats in the MQSD group received oral gavage of MQSD extract (2.0 g/kg) twice daily for seven consecutive days, while the saline group received an equal volume of normal saline. On Day 7, under anaesthesia with 10% chloral hydrate (0.3 mL/100 g), blood was collected via the abdominal aorta. Serum was isolated by centrifugation at 3000 rpm for 15 min and stored at −80°C. The major components of the MQSD extract were analysed using HPLC (as previously reported by [28]) to ensure batch‐to‐batch consistency and reproducibility. Representative chromatograms are shown in Figure 1, and detailed concentrations are provided in Table 2.

**TABLE 2:** Main compounds of MQSD.

No.	Compounds	Chemical forumula	Contents (mg/g)
1	Cimicifugoside	C_37_H_54_O_11_	3.77
2	Compound Glycyrrhizin	C_42_H_62_O_16_	17.75
3	5‐Hydroxymethylfurfural	C_6_H_6_O_3_	0.49
4	Osthole	C_15_H_16_O_3_	7.29
5	Notopterol	C_21_H_22_O_5_	76.12
6	Oxypeucedan hydrate	C_16_H_16_O_6_	3.53

### 2.14 qRT‐PCR

The qRT‐PCR procedure was conducted as per established protocols [28]. The primer sequences utilised are delineated in Table 3.

**TABLE 3:** Primer sequence.

Gene	Forward	Reverse
GAPDH	TGTGTCCGTCGTGGATCTGA	TTGCTGTTGAAGTCGCAGGAG
COL2A1	CCAGATTGAGAGCATCCGCA	ACTTTCATGGCGTCCAAGGT
SOX9	CCACCCCGATTACAAGTACCAG	CAGCGCCTTGAAGATAGCAT
MMP13	TTCTGGTCTTCTGGCACACG	TTGTAGCCTTTGGAACTGCTTG
ADAMTS5	GCTAAGGGCACAGGCTACTATG	CCGTCACATCCAGTTCTCACA
AKT1	CCTCAAGAACGATGGCACCT	TGCAGGCAGCGGATGATAAA
GPX4	GCCTGGATAAGTACAGGGGTT	CATGCAGATCGACTAGCTGAG

### 2.15 Western Blotting

The protocol has been described previously [28]. The primary antibodies used are described in the reagents section.

### 2.16 Animal Experiments

The animal experiments conducted in this study were approved by the Ethics Committee of Zhejiang Chinese Medicine University (Approval No. IACUC‐ 20240603‐21). C57BL/6 mice (male, SPF grade, 16‐24 g, 6–8 weeks of age, Supplier: Hangzhou Qizhen Co.) and SD rats (male, SPF grade, 50–250 g, 3–7 weeks of age, Supplier: Shanghai BK Co.) were used. Animal experiments were carried out at the Animal Experiment Center of Zhejiang Chinese Medicine University (Hangzhou, China), under the licence for the use of experimental animals [SYXK (Zhe)2021–0012]. The mice and rats utilised in this study were housed under standard husbandry conditions, provided access to food and water ad libitum, and kept in an air‐controlled room at 25°C ± 1°C with a 12‐h light/dark cycle. All animals were anaesthetised using intraperitoneal injection of Sutai anaesthetic, and euthanasia was performed using carbon dioxide.

### 2.17 OA Mice Model and Treatment Protocol

OA modelling was established following the previously disclosed protocol [28]. Animals were categorised into the following groups: SHAM, DMM, MQSD low dose (0.14 g/kg), MQSD medium dose (0.28 g/kg), and MQSD high dose (0.56 g/kg), with six mice allocated to each group. MQSD gavage treatment was administered twice daily for a total duration of 4 weeks. During this period, sham and model groups received equal volumes of saline gavage to maintain consistency in administration protocols.

### 2.18 Micro‐CT Scanning

Micro‐CT scans were performed in accordance with the previously disclosed protocol [28].

### 2.19 Saffron‐O and Fast Green Staining

Paraffin sections of mouse knee joints were dewaxed and rehydrated. Fresh Weigert staining solution was prepared according to the manufacturer's instructions and drop‐stained onto the sections for 3 min. The sections were then washed three times with pure water for 3 min each. Subsequently, they were drop‐stained with acidic differentiation solution for 15 s, followed by another three washes with pure water for 3 min each. The sections were then immersed in Solid Green staining solution for 3 min. After a quick wash with weak acid solution, the sections were immersed in saffron staining solution for 5 min. Subsequently, gradient alcohol dehydration and xylene transparency were performed, followed by sealing with neutral gum. Images were captured using a digital pathology slide scanning system (Hamamatsu, Japan).

### 2.20 Immunohistochemistry (IHC)

Paraffin sections of mouse knee joints were prepared, followed by dewaxing and rehydration. Antigen retrieval was performed, and the sections were subsequently sealed. Primary antibody was applied and incubated overnight at 4°C. Enhanced enzyme‐labelled goat anti‐rabbit IgG polymer was then added dropwise and incubated for 20 min at 37°C in an oven. DAB (3,3′‐diaminobenzidine) was utilised to develop the colour. The sections were washed sequentially by immersion in haematoxylin, hydrochloric alcohol, ammonia and PBS. After drying, they were made translucent in xylene and sealed with neutral gum. Images were captured using a digital pathology slide scanning system (Hamamatsu, Japan).

### 2.21 Network Pharmacology Analysis

First, active ingredients were screened from the TCMSP database [29] based on DL value ≥ 0.18 and OB value ≥ 30%, and from the BATMAN‐TCM database [30] based on Score > 20. The identified active ingredients were combined, and their corresponding target points were obtained to establish the final target points of MQSD active ingredients. Next, the OMIM [31] and Genecards databases [32] were queried using the keyword “Osteoarthritis” to retrieve OA disease targets.

The intersection of TCM targets and disease targets was visualised as a Venn diagram using pyvenn to identify drug‐disease common targets. Subsequently, a disease TCM‐target map was constructed using Cytoscape, with green circular nodes representing TCMs and yellow prismatic nodes representing targets intersecting with diseases. To elucidate the function of candidate targets, GO and KEGG enrichment analyses were performed using clusterProfiler (version 4.10.0). Significant enrichment results were selected based on a criterion of *p*_adjust_ < 0.05 and count value > 1. The top 10 GO terms and top 20 KEGG pathways were visualised as histograms and bubble diagrams, respectively. Additionally, the top 20 KEGG pathways were visualised using emap according to the JC coefficient.

### 2.22 Molecular Docking

Molecular docking was conducted for 5‐hydroxymethylfurfural, oxypeucedan hydrate, Cimicifugoside, Notopterol, and their respective protein receptors AKT1, PIK3CD and GPX4. Firstly, 3D structures of the active ingredients were obtained by searching their chemical names in English on the PubChem database (https://pubchem.ncbi.nlm.nih.gov/) and saving the files in SDF format. Subsequently, the core target protein IDs were retrieved from the UniProt database (https://www.uniprot.org/) using the keyword of the core target gene name. The 3D structure files of the core target proteins were then downloaded from the Protein Data Bank (PDB) and saved in PDB format. Water molecules and small molecule ligands were removed from the protein structures using Pymol 2.5 open source software, and the modified files were saved as PDB files. The active pocket sites of the protein receptors were predicted using ProteinsPlus (https://proteins.plus/), and molecular docking was performed using AutoDock Vina v1.2.5. Each docking was performed 20 times, and the conformation with the lowest free energy was selected as the optimal result. The optimal model pdb files were visualised using PLIP with Pymol.

### 2.23 Statistical Analysis

Data analysis was conducted using GraphPad Prism 9 software. Normally distributed measurements were presented as mean ± standard deviation (mean ± SD), and comparisons between groups were analysed using the *t*‐test. One‐way analysis of variance (ANOVA) with Tukey's multiple comparison test was employed to assess the statistical significance of group comparisons. A *p*‐value less than 0.05 was considered statistically significant.

## 3 Results

### 3.1 Quality Control and Chemical Profiling of MQSD

The MQSD was supplied by the First Affiliated Hospital of Zhejiang Chinese Medicine University (Hangzhou, Zhejiang, China). The composition of Chinese medicinal herbs in MQSD is detailed in Table 1. This traditional concoction was initially brewed as a decoction, subsequently concentrated, and transformed into dry powder via freeze‐drying. The qualitative and quantitative assessment of MQSD's principal chemical constituents was conducted employing High‐Performance Liquid Chromatography (HPLC). Given the differential light absorption characteristics of certain compounds, their analysis was captured through three distinct chromatograms, as shown in Figure 1. The concentrations of these components are listed in Table 2.

### 3.2 MQSD Manifests a Bifunctional Impact on Chondrocytes, Attenuating IL‐1β‐Triggered Apoptosis and Concurrently Enhancing Their Proliferative and Differentiation Potential

**FIGURE 2:**
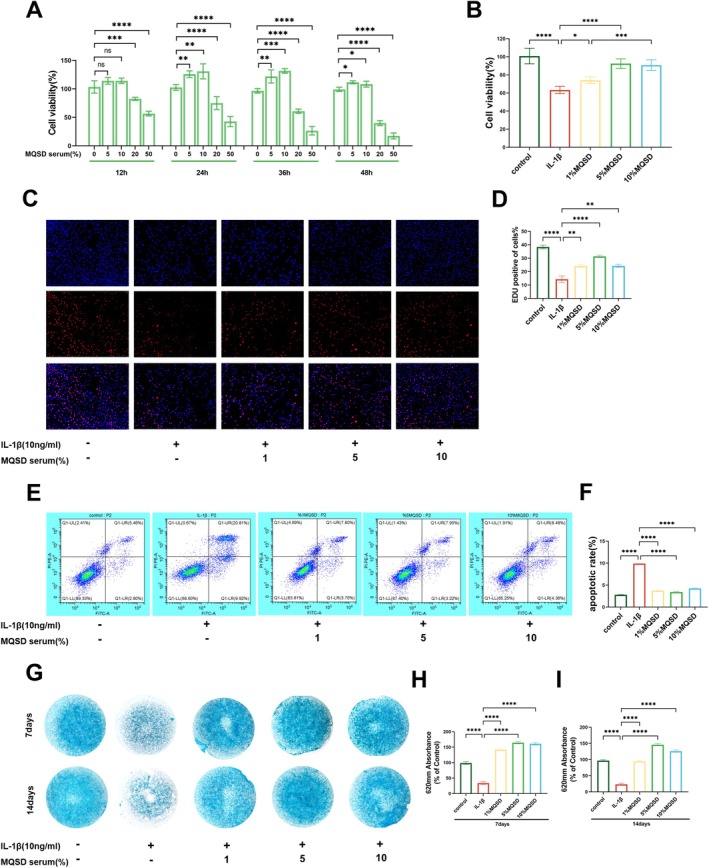
MQSD enhances IL‐1β‐induced chondrocyte viability and differentiation capacity. (A) Gradient concentration of MQSD was applied to chondrocytes for 12 h, 24 h, 36 h, 48 h and its effect on cell viability was detected by CCK8. (B) CCK8, (C, D) EDU, and (E, F) apoptosis of chondrocytes cultured with IL‐1β (10 ng/mL) for 24 h. (G–I) Alcian Blue staining of chondrocytes cultured with IL‐1β for 24 h. All data represent mean ± SEM. *p < 0.05, **p < 0.01, ***p < 0.001 and ****p < 0.0001 by one‐way ANOVA.

To ascertain the ideal concentration and treatment duration of MQSD, chondrocytes underwent exposure to various MQSD‐serum concentrations (0%, 5%, 10%, 20% and 50%) across time intervals of 12, 24, 36 and 48 h. Cell viability, evaluated via the CCK‐8 assay, exhibited a dose‐responsive enhancement within the 0%–10% MQSD concentration span, notably achieving peak efficacy at 24 h (Figure 2A). Follow‐up investigations adopted 1%, 5%, and 10% concentration gradients from this spectrum. Remarkably, even under IL‐1β (10 ng/mL) stimulation, pre‐treatment with MQSD preserved its cell viability augmentation effect, with 5% concentration yielding the most pronounced benefit (Figure 2B). In vitro models of OA‐induced inflammation, complemented by EDU incorporation and flow cytometry analyses, indicated that MQSD notably fostered chondrocyte proliferation while curbing apoptosis (Figure 2C–F). Furthermore, Alcian Blue staining underscored that MQSD treatment culminated in enhanced ECM production by chondrocytes at the end of the stimulation period, as well as after 7 and 14 days within microsphere culture environments (Figure 2G–I).

### 3.3 Cyberpharmacological Evaluation of MQSD

**FIGURE 3:**
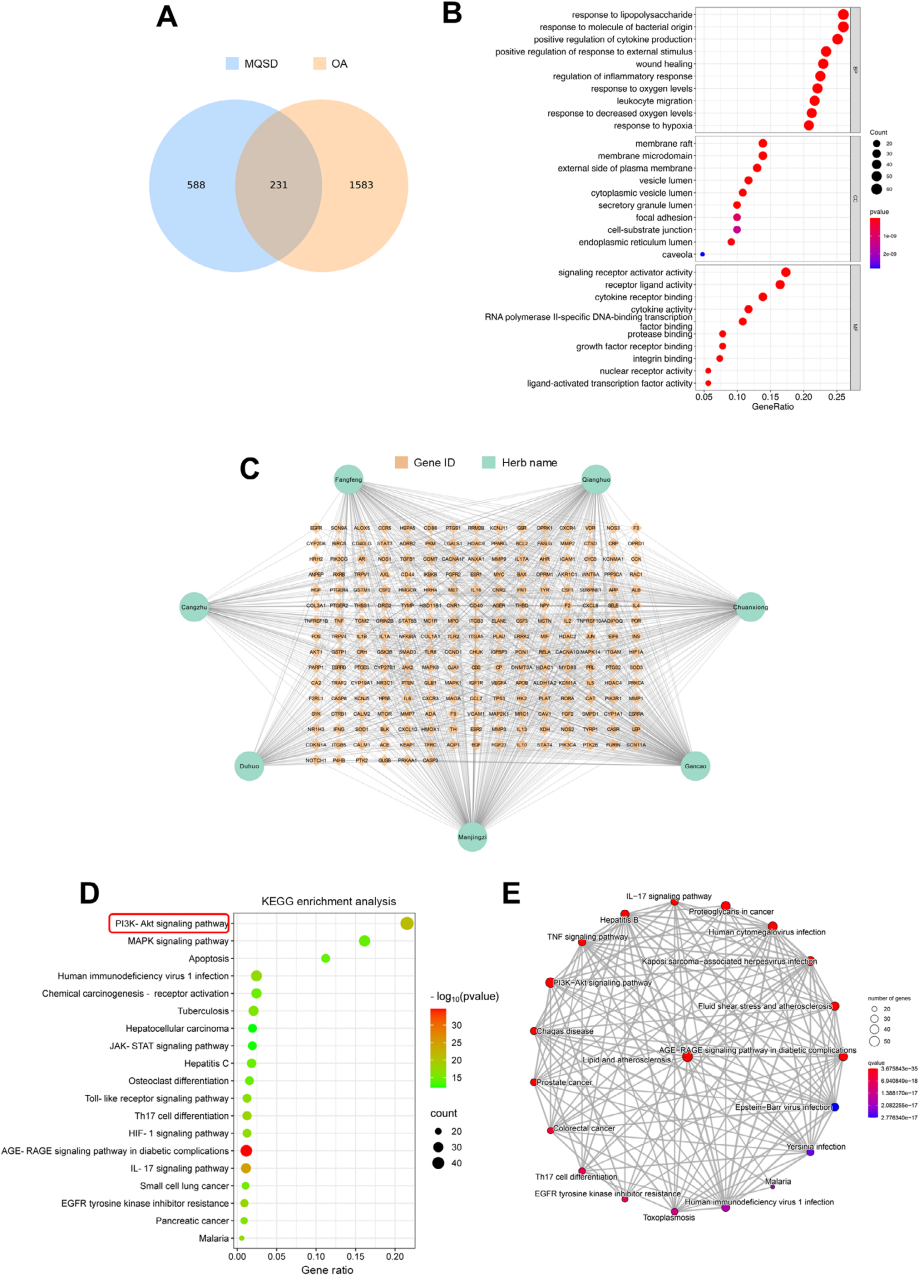
Pharmacological prediction of active ingredients and target network of MQSD in OA treatment. (A) Venn diagram illustrating 231 intersections between MQSD and OA. (B) Histogram depicting GO enrichment analysis for biological processes (BP), cellular components (CC), and molecular functions (MF). (C) Network map of herbal components‐OA targets constructed using Cytoscape. (D) Bubble diagram representing KEGG enrichment analysis results. (E) Visualisation network diagram of KEGG pathways constructed using emap.

Upon analysis using the TCMSP and BATMAN‐TCM databases, 746 active components were identified. These components' associated targets were subsequently ascertained, uncovering 819 targets linked to MQSD's active compounds. Further research utilising the OMIM and Genecards databases identified 1814 targets relevant to OA. Analysis of these data sets found 231 targets common between MQSD and OA (Figure 3A). A network map created with Cytoscape depicted the overlap of seven herbal elements of MQSD with OA‐related targets (Figure 3C). GO enrichment analysis yielded 3068 biological process (BP) entries, 114 cellular component (CC) entries, and 206 molecular function (MF) entries, spotlighting key pathways like lipopolysaccharide response, oxygen level response, and inflammatory response regulation (Figure 3B). KEGG pathway enrichment identified 189 significant pathways, highlighting that MQSD's active ingredients primarily influence OA via pathways such as PI3K‐Akt, IL‐17 and TNF signalling (Figure 3D). The top 20 KEGG pathways were visualised using an emap to showcase their interconnectivity (Figure 3E). These results collectively imply MQSD's potential action on OA through these identified pathways.

### 3.4 MQSD'S Suppression of Ferroptosis Regulates Anabolic‐Catabolic Equilibrium in Chondrocytes

**FIGURE 4:**
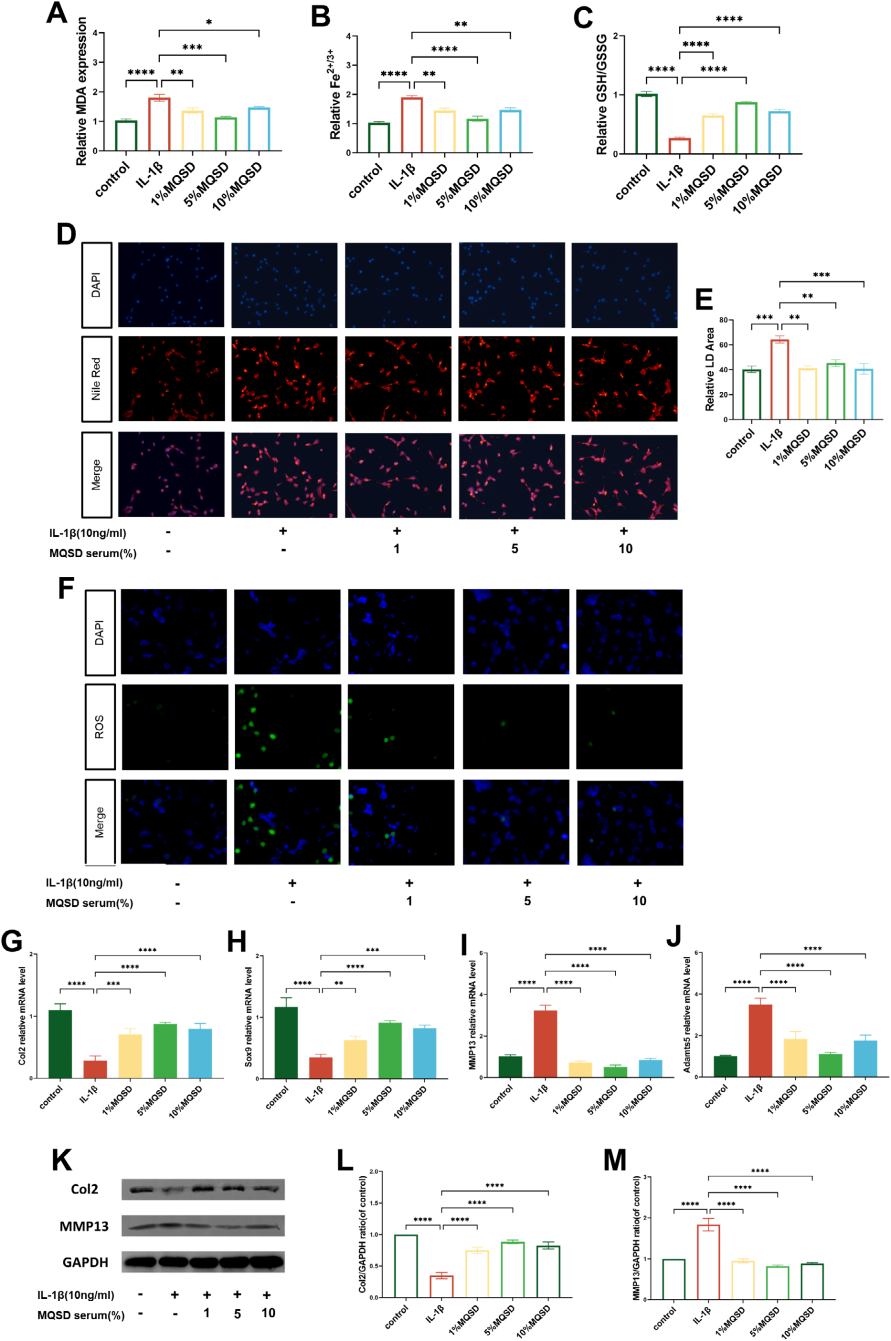
MQSD modulates IL‐1β‐induced anabolic‐catabolic homeostasis and inhibits ferroptosis in chondrocytes. (A–C) MDA, Fe^2+/3+^ and GSH/GSSG levels were detected by the relative Assay kits. (D, E) Nile Red staining of chondrocytes cultured with IL‐1β for 24 h. (F) ROS of chondrocytes analysed by fluorescence microscope. (G–J) qRT‐PCR analysis of Col2, Sox9, MMP13 and Adamts5 relative mRNA expression by chondrocytes cultured with IL‐1β for 24 h. (K–M) Western blot analysis of Col2 and MMP13 expression by chondrocytes cultured with IL‐1β for 24 h. All data represent mean ± SEM. *p < 0.05, **p < 0.01, ***p < 0.001 and ****p < 0.0001 by one‐way ANOVA.

Upon MQSD pretreatment and subsequent IL‐1β challenge, chondrocytes' ferroptosis‐associated phenotypes were assessed. The IL‐1β group displayed a marked increase in MDA and iron levels, coupled with a diminished GSH/GSSG ratio. In contrast, the MQSD‐treated group showed a significant decrease in MDA and iron levels and an elevated GSH/GSSG ratio (Figure 4A–C). Lipid droplet staining revealed an increase in lipid droplet accumulation in the IL‐1β group compared to MQSD‐treated cells (Figure 4D,E), whereas ROS levels were lower in the MQSD group, indicating reduced oxidative stress (Figure 4F). qRT‐PCR analysis revealed that MQSD upregulated anabolic markers such as Col2 and Sox9 and downregulated catabolic markers like MMP13 and Adamts5 (Figure 4G–J). These results were supported by western blot analysis, which showed increased Col2 and decreased MMP13 levels in the MQSD group (Figure 4K–M).

**FIGURE 5:**
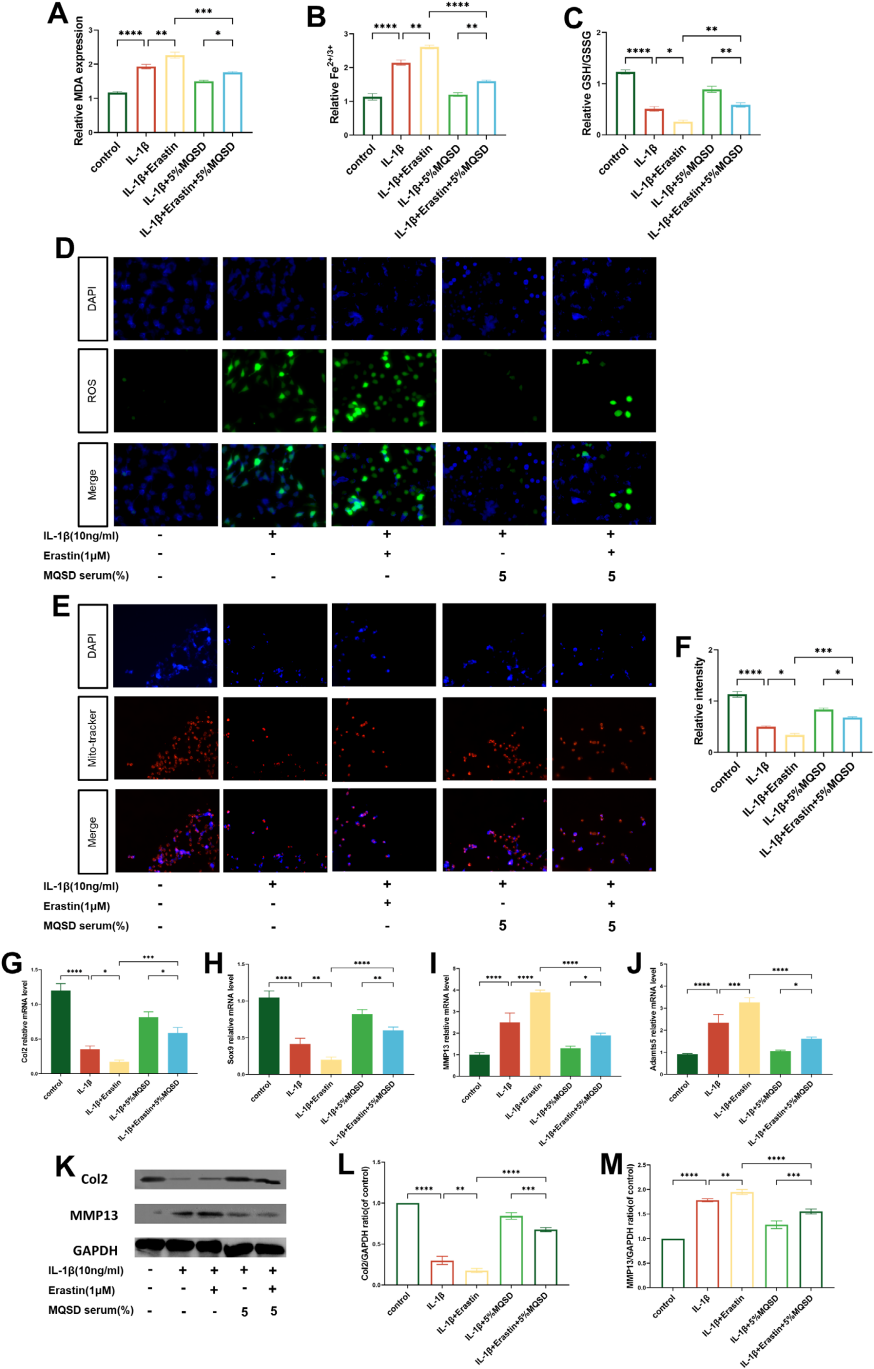
MQSD rescued the anabolic catabolic phenotype of chondrocytes stimulated by the ferroptosis agonist Erastin. (A–C) MDA, Fe^2+/3+^ and GSH/GSSG levels were detected by the relative Assay kits. (D) ROS of chondrocytes analysed by fluorescence microscope. (E, F) Mito‐tracker staining of chondrocytes analysed by fluorescence microscope. (G–J) qRT‐PCR analysis of Col2, Sox9, MMP13 and Adamts5 relative mRNA expression by chondrocytes cultured with IL‐1β (10 ng/mL) and erastin (1 μM). (K–M) Western blot of Col2 and MMP13 in chondrocytes treated with IL‐1β together with erastin. All data represent mean ± SEM. *p < 0.05, **p < 0.01, ***p < 0.001 and ****p < 0.0001 by one‐way ANOVA.

**FIGURE 6:**
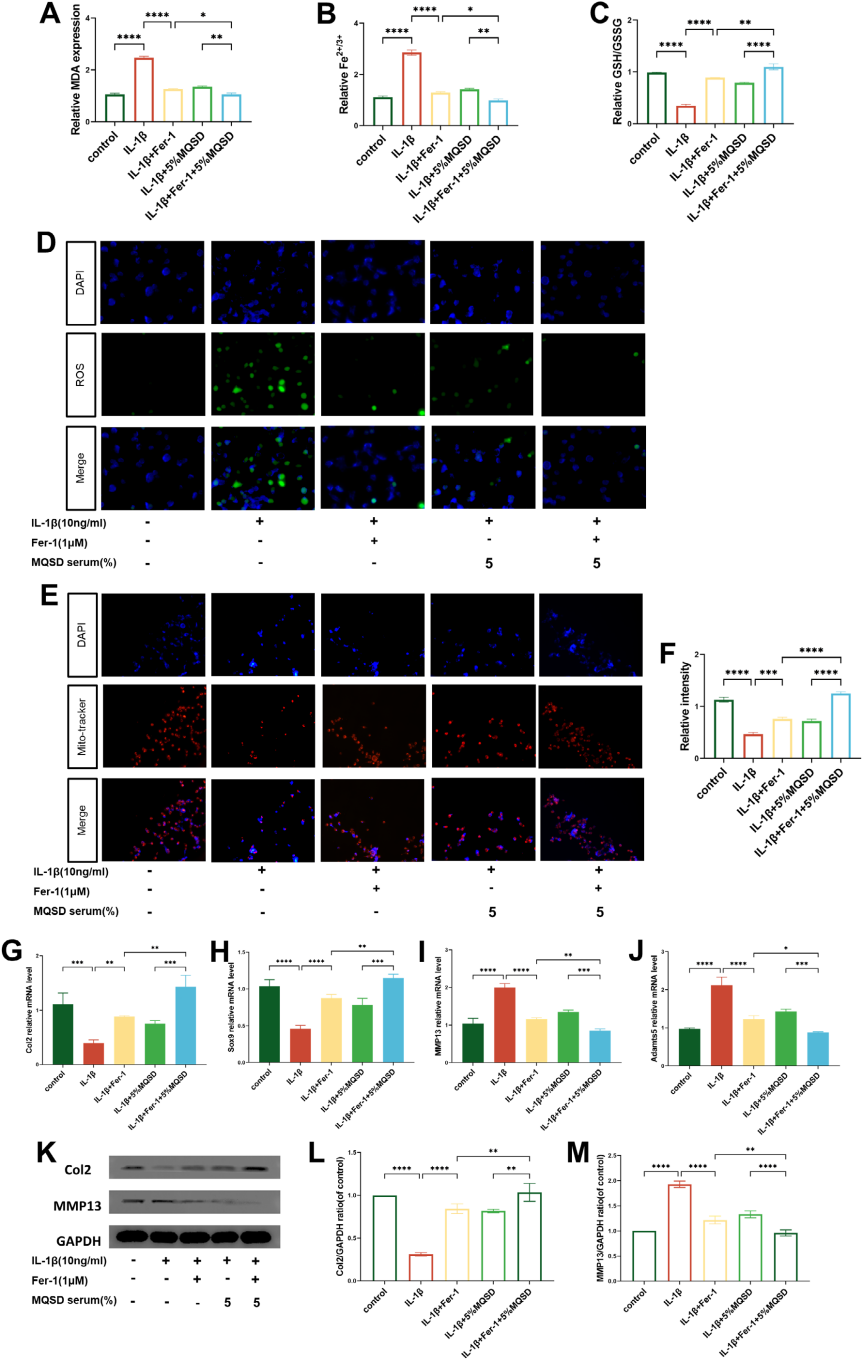
MQSD modulates chondrocyte anabolic catabolic phenotype with similar effects to the ferroptosis inhibitor Ferrostatin‐1. (A–C) MDA, Fe^2+/3+^ and GSH/GSSG levels were detected by the relative Assay kits. (D) ROS of chondrocytes analysed by fluorescence microscope. (E, F) Mito‐tracker staining of chondrocytes analysed by fluorescence microscope. (G–J) qRT‐PCR analysis of Col2, Sox9, MMP13 and Adamts5 relative mRNA expression by chondrocytes cultured with IL‐1β (10 ng/mL) and Ferrostatin‐1(1 μM). (K–M) Western blot of Col2 and MMP13 in chondrocytes treated with IL‐1β together with Ferrostatin‐1. All data represent mean ± SEM. *p < 0.05, **p < 0.01, ***p < 0.001 and ****p < 0.0001 by one‐way ANOVA.

Further treatment with the ferroptosis inducer Erastin and inhibitor Fer‐1 delineated MQSD's correlation with ferroptosis. Erastin treatment elevated MDA and iron levels, boosted ROS, and lowered the GSH/GSSG ratio (Figure 5A–D), effects that were mitigated by MQSD co‐treatment. Mito‐tracker staining showed fewer mitochondria post‐Erastin, a trend reversed by MQSD (Figure 5E,F). qRT‐PCR showed MQSD enhanced Col2 and Sox9 expression while reducing MMP13 and Adamts5 levels (Figure 5G–J), a finding echoed in Western blot results. With Fer‐1 co‐administration, both MQSD and Fer‐1 lessened MDA and iron levels, curtailed ROS, and improved the GSH/GSSG ratio, along with mitochondrial count enhancement (Figure 6A–F). qRT‐PCR confirmed both treatments upregulated Col2 and Sox9 while downregulating MMP13 and Adamts5 (Figure 6G–J), with western blot analyses validating these effects. Collectively, these outcomes strongly suggest MQSD's role in OA mitigation via ferroptosis inhibition in chondrocytes.

### 3.5 MQSD Attenuates Chondrocyte Ferroptosis by Suppressing the PI3K/Akt Signalling Pathway

**FIGURE 7:**
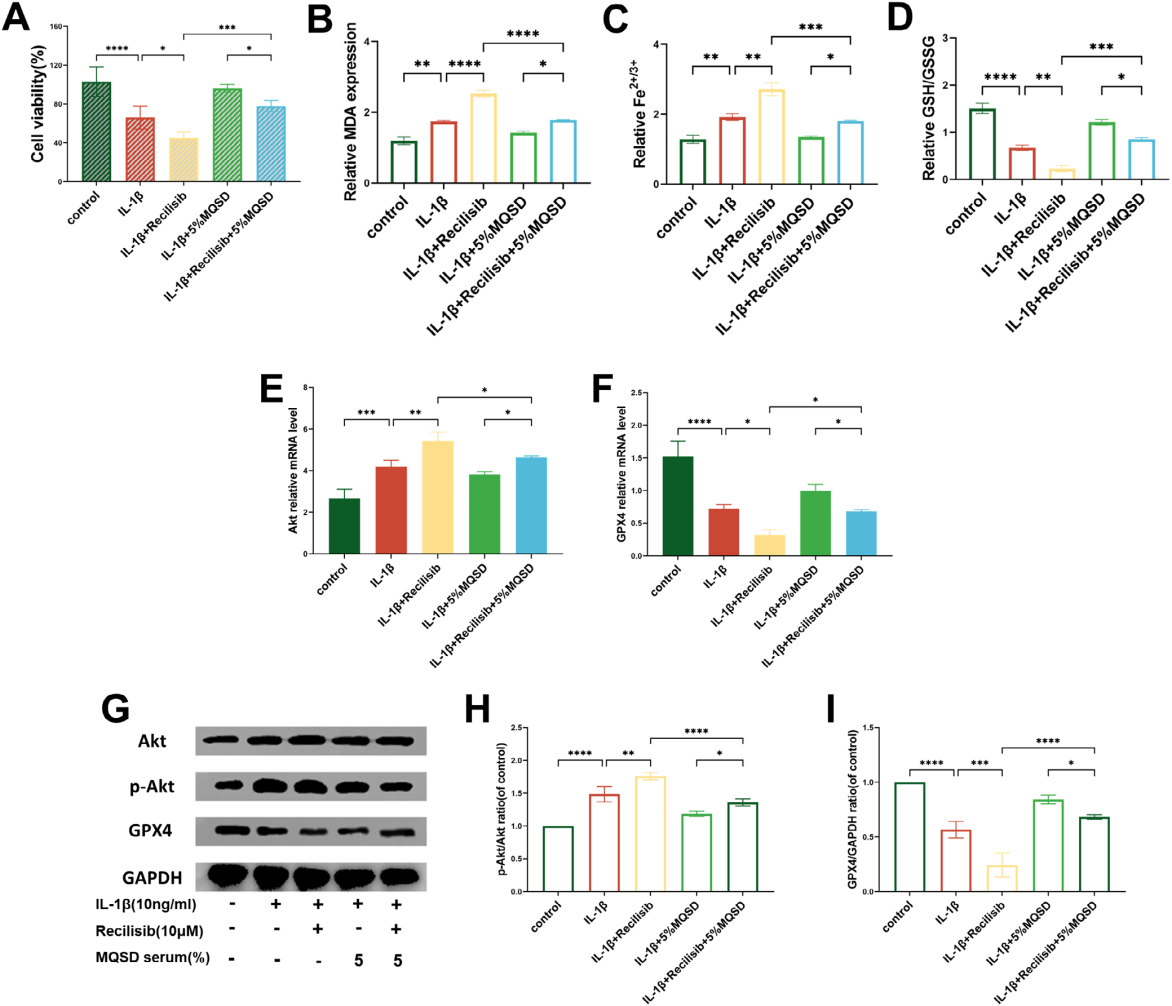
MQSD suppresses ferroptosis by inhibiting Akt phosphorylation and rescues the ferroptosis phenotype in PI3K/Akt agonist Recilisib‐stimulated chondrocytes. (A) CCK8 of chondrocytes treated with IL‐1β (10 ng/mL) together with Recilisib (10 μM).(B–D) MDA, Fe^2+/3+^ and GSH/GSSG levels were detected by the relative Assay kits. (E, F) qRT‐PCR analysis of Akt and GPX4 relative mRNA expression by chondrocytes cultured with IL‐1β (10 ng/mL) and Recilisib (10 μM). (G–I) Western blot of Akt, p‐Akt and GPX4 in chondrocytes treated with IL‐1β together with Recilisib. All data represent mean ± SEM. *p < 0.05, **p < 0.01, ***p < 0.001 and ****p < 0.0001 by one‐way ANOVA.

Our network pharmacological analysis suggested that the PI3K/Akt signalling pathway could be a key target of MQSD in OA treatment. To elucidate this mechanism, we employed Recilisib, an activator of the PI3K/Akt pathway, and LY294002, a PI3K/Akt pathway inhibitor. Initially, chondrocytes were treated with Recilisib (10 μM). The results revealed that MQSD treatment significantly enhanced chondrocyte viability (Figure 7A), reduced MDA levels (Figure 7B) and iron levels (Figure 7C) and increased the GSH/GSSG ratio (Figure 7D). qRT‐PCR analysis demonstrated a decrease in Akt expression and a significant increase in GPX4 expression upon MQSD treatment (Figure 7E,F). Correspondingly, western blot analysis showed a reduction in phosphorylated AKT (p‐AKT) expression and an increase in GPX4 expression in chondrocytes following MQSD treatment (Figure 7G–I).

**FIGURE 8:**
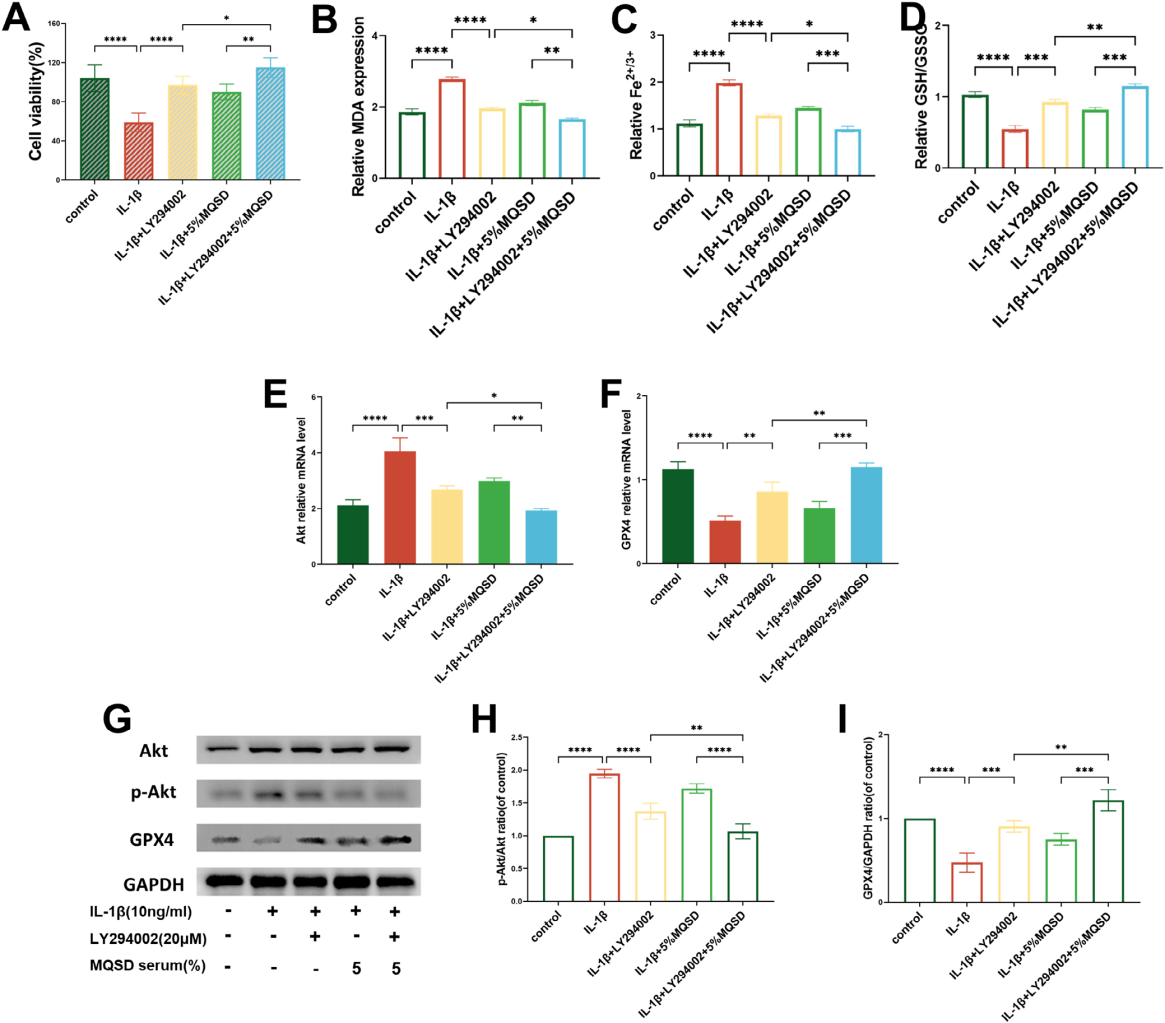
MQSD and PI3K/Akt inhibitor LY294002 are similarly effective in inhibiting ferroptosis phenotype in chondrocytes. (A) CCK8 of chondrocytes treated with IL‐1β (10 ng/mL) together with LY294002 (20 μM).(B–D) MDA, Fe^2+/3+^ and GSH/GSSG levels were detected by the relative Assay kits. (E, F) qRT‐PCR analysis of Akt and GPX4 relative mRNA expression by chondrocytes cultured with IL‐1β and LY294002. (G–I) Western blot of Akt, p‐Akt and GPX4 in chondrocytes treated with IL‐1β together with LY294002. All data represent mean ± SEM. *p < 0.05, **p < 0.01, ***p < 0.001 and ****p < 0.0001 by one‐way ANOVA.

Furthermore, both LY294002 (20 μM) and MQSD treatments exhibited similar effects. Both treatments led to increased chondrocyte viability (Figure 8A), decreased MDA levels (Figure 8B) and iron levels (Figure 8C), and enhanced GSH/GSSG expression (Figure 8D). qRT‐PCR results revealed that both LY294002 and MQSD downregulated Akt expression while promoting GPX4 expression (Figure 8E,F). This was further supported by western blot analysis, which showed inhibition of p‐AKT expression and elevation of GPX4 expression with both LY294002 and MQSD treatments (Figure 8G–I). These findings suggest that MQSD's ability to mitigate chondrocyte ferroptosis may be attributed to its modulation of the PI3K/Akt signalling pathway.

### 3.6 In Vivo Experiments Validating MQSD'S Efficacy and Mechanism of Action

**FIGURE 9:**
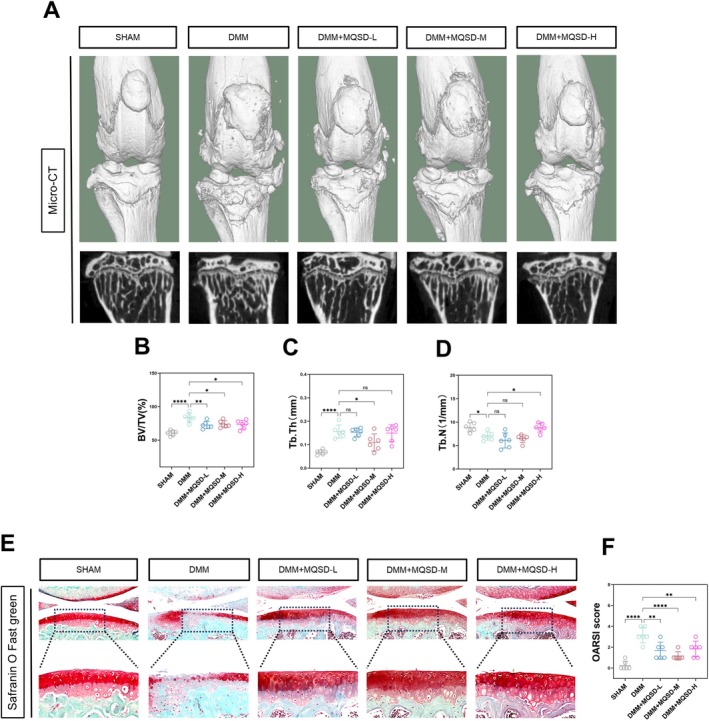
MQSD postpones OA progression in the mouse DMM model. (A) Micro‐CT of C57BL/6 mice treated with different concentrations of MQSD.(0.14, 0.28, and 0.56 g/kg). (B–D) Quantitative results of bone volume fraction (BV/TV), bone trabecular thickness (Tb.Th), and trabecular number (Tb.N). (E, F) Representative images of Safranin O Fast green staining and OARSI score. All data represent mean ± SEM. *p < 0.05, **p < 0.01, ***p < 0.001 and ****p < 0.0001 by one‐way ANOVA.

In our in vitro experiments, we established that MQSD delays the progression of OA by impeding chondrocyte ferroptosis through the PI3K/Akt signalling axis. To confirm this conclusion, we conducted in vivo experiments using a mouse OA model to determine if MQSD exhibited the same efficacy in vivo. Micro‐CT results revealed marked degeneration in the knee joints of mice in the DMM group, characterised by partial dislocation of the medial meniscus, extensive osteophyte formation around the medial knee compartment, and significant sclerosis of the subchondral bone of the medial tibial plateau articular surface. In contrast, mice treated with MQSD showed a significant slowing down of OA progression. Compared to the DMM group, the MQSD‐treated groups exhibited significantly fewer bone remnants, with higher subchondral bone porosity and lower degrees of sclerosis (Figure 9A). Quantitative analysis of bone volume fraction (BV/TV) (Figure 9B), bone trabecular thickness (Tb.Th) (Figure 9C), and number of bone trabeculae (Tb.N) are depicted in Figure 9D.

Subsequently, we examined the mouse knee joints through pathological sectioning and examination. Safranin O‐Fast Green staining revealed a significant reduction in cartilage‐specific staining, decreased cartilage thickness, and irregular cartilage surfaces in the DMM group. Conversely, the MQSD‐treated groups displayed smoother and thicker cartilage surfaces (Figure 9E). Quantitative results of the Osteoarthritis Research Society International (OARSI) scoring system for articular cartilage pathology sections are shown in Figure 9F.

**FIGURE 10:**
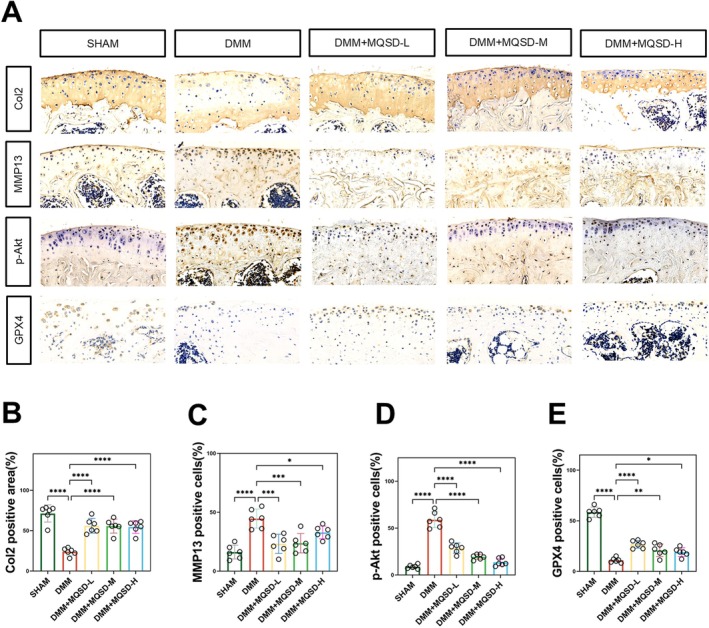
PI3K/Akt/GPX4 mediates the chondroprotective effect of MQSD in vivo. (A) Immunohistochemical staining of Col2, MMP13, p‐Akt, and GPX4 in C57BL/6 mice treated with different concentrations of MQSD (0.14, 0.28, and 0.56 g/kg). (B–E) The above quantitative immunohistochemical analysis results. All data represent mean ± SEM. *p < 0.05, **p < 0.01, ***p < 0.001, and ****p < 0.0001 by one‐way ANOVA.

To further validate MQSD's efficacy and mechanism at the in vivo protein level, we conducted IHC assays. The results demonstrated a significant decrease in the expression of Col2, the primary component maintaining the elasticity and resistance to deformation of articular cartilage, in mice from the DMM group, along with an increase in MMP13 expression, known for its cartilage‐degrading properties. However, upon treatment with MQSD, the stained area of Col2 in the pathological sections of mice significantly increased, while MMP13‐positive expression was markedly suppressed. Mechanistically, our examination of p‐Akt and GPX4 showed results consistent with our in vitro experiments. MQSD notably inhibited the phosphorylation of Akt proteins and promoted the expression of GPX4, a pivotal regulator of anti‐ferroptosis, in mice (Figure 10A). The results of the above quantitative analysis are shown in Figure 10B–E. In summary, MQSD demonstrated continued anti‐OA efficacy in vivo, likely achieved by regulating iron death following Akt protein phosphorylation inhibition.

### 3.7 Analysing the Molecular Basis of MQSD'S Regulation of PI3K/Akt‐Associated Ferroptosis in the Context of Anti‐OA Effects Through Molecular Docking Technique

**FIGURE 11:**
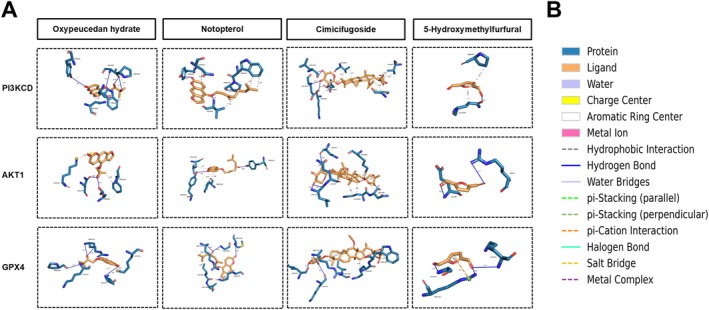
Analysis of MQSD anti‐OA and PI3K/Akt/GPX4‐related ferroptosis active ingredients revealed by molecular docking technique. (A) Legend of visualisation of molecular docking results. Oxypeucedan hydrate, Notopterol, Cimicifugoside, and 5‐Hydroxymethylfurfural were docked with PI3KCD, AKT1, and GPX4, respectively. (B) Interpretation of colours in the molecular docking visualisation legend.

In order to delve deeper into MQSD's mechanism of inhibiting ferroptosis in chondrocytes by modulating the PI3K/Akt signalling pathway to mitigate OA progression, we conducted molecular docking studies of MQSD's principal components with three pivotal proteins: PI3KCD, AKT1 and GPX4, respectively. The protein structures of PI3KCD (6PYR), AKT1 (PDBID:6HHJ) and GPX4 (PDBID:2OBI) were retrieved from the RSCB PDB database (https://www.rcsb.org/). Docking simulations were executed using AutoDock Vina v1.2.5. The outcomes of the optimal model exhibited a binding energy of less than −5.0 kcal/mol, signifying favourable interactions between the target proteins and the active constituents. Lower binding energies suggest a higher likelihood of binding, while an increased number of hydrogen bonds indicates a stronger affinity. The molecular docking results were depicted visually, as illustrated in Figure 11A,B, suggesting that oxypeucedan hydrate, Notopterol and Cimicifugoside, the principal active ingredients of MQSD, could modulate PI3K/Akt‐associated ferroptosis.

## 4 Discussion

This research aimed to explore the efficacy and molecular underpinnings of MQSD, a celebrated TCM formulation known for its anti‐inflammatory and analgesic effects, in mitigating OA progression. Utilising HPLC for MQSD quality assurance, we combined network pharmacology and molecular docking approaches with extensive in vitro and in vivo studies to validate our theoretical constructs and empirical observations.

To establish a quantitative‐effectiveness relationship between MQSD and chondrocyte protection in vitro, we administered varying concentrations of MQSD‐containing serum to chondrocytes and monitored its efficacy at multiple time points. While prior research [11] has highlighted MQSD's notable anti‐inflammatory and analgesic effects in murine models, we sought to ascertain whether its chondrocyte‐protective effects were mediated through anti‐inflammatory pathways. To delve deeper, we established an IL‐1β‐induced chondrocyte inflammation model in vitro. In this model, pre‐treatment with MQSD significantly attenuated chondrocyte apoptosis rates, as evidenced by reduced apoptosis compared to controls. Additionally, MQSD administration was associated with enhanced chondrocyte proliferation, as demonstrated by increased EDU incorporation. Moreover, Alcian Blue assay revealed heightened secretion of extracellular matrix components by chondrocytes in the presence of MQSD. These findings collectively underscore the efficacy of MQSD in fostering both the quantity and quality of chondrocyte growth. Consequently, we embarked on further investigations into chondrocyte death and anabolic catabolism, crucial homeostatic mechanisms implicated in OA progression.

The pathogenesis of OA predominantly stems from the disruption of cartilage homeostasis, characterised by aberrant chondrocyte viability and metabolic imbalances triggered by multifactorial influences [33]. Despite the classification of glucosamine and chondroitin sulfate as “chondroprotective agents,” their clinical efficacy remains contentious, with inconclusive evidence regarding their ability to modulate cartilage metabolism and impede extracellular matrix degradation [34, 35]. Nonsteroidal antiinflammatory drugs (NSAIDs), primary pharmacotherapeutic options for OA, alleviate inflammatory pain but lack direct chondroprotective effects, while protracted usage heightens the susceptibility to cardiovascular, cerebrovascular, and gastrointestinal complications [36]. Patients subjected to minimally invasive procedures such as arthroscopic debridement surgery for OA did not exhibit superior outcomes compared to those receiving placebo interventions, failing to achieve disease remission [37]. Total knee replacement, while efficacious in symptom management, necessitates complete cartilage removal and poses considerable financial and structural ramifications [38]. Consequently, the absence of a universally recognised therapeutic intervention underscores the relevance of exploring novel strategies, such as MQSD, to address the intricate pathophysiology of OA.

QSD, originating from the classical Chinese medical text spleen and stomach, is extensively employed clinically to address inflammatory pain‐related ailments such as rheumatoid arthritis and ankylosing spondylitis [39, 40]. In order to enhance its efficacy in treating OA, we refined this formulation by incorporating Atractylodes lancea DC (Cangzhu), an herb renowned for its anti‐inflammatory properties [41]. Analysis of the HPLC fingerprints of MQSD, comprising eight herbs, revealed the presence of six active ingredients, namely Cimicifugoside, compound Glycyrrhizin, 5‐hydroxymethylfurfural, Osthole, Notopterol, and oxypeucedan hydrate. Notably, 5‐hydroxymethylfurfural, Osthole, Notopterol, and oxypeucedan hydrate have been identified as modulators of the biological functions of the PI3K/Akt signalling axis, a finding supported by our network pharmacology predictions. Specifically, the Yinxieling optimization formula containing 5‐Hydroxymethylfurfural has demonstrated efficacy in modulating inflammatory factors via the PI3K/Akt signalling pathway for the treatment of psoriasis [42]. Osthole exhibits regulatory effects on the PI3K/Akt pathway for cancer treatment [43], while Notopterol has been shown to regulate the PI3K/Akt signalling pathway to confer chondroprotective effects [13]. Given the potential of MQSD, containing these active components, to protect cartilage and impede OA progression, it warrants further investigation into its mechanism of action. Our in vitro investigations revealed that MQSD can modulate the synthesis and catabolism balance of inflammatory chondrocytes, enhance chondrocyte proliferation, and mitigate apoptosis. Furthermore, our in vivo studies in mice demonstrated that MQSD could mitigate osteochondral redundancy and subchondral osteosclerosis, thereby protecting cartilage and delaying OA progression. In conclusion, MQSD emerges as a promising therapeutic avenue for addressing OA.

Numerous studies have revealed the involvement of ferroptosis in the mechanism of cartilage homeostasis in OA. Inflammatory stimuli and oxidative stress damage acting on chondrocytes will trigger the dysregulation of iron ion homeostasis, which in turn will lead to excessive lipid peroxide accumulation [44, 45]. Then, we hypothesised that MQSD, which has anti‐inflammatory and antioxidant effects, can counteract ferroptosis in the treatment of OA, which was confirmed in our experimental validation, as shown by the fact that MQSD decreased the levels of iron ions and MDA in chondrocytes stimulated by IL‐1β or the ferroptosis agonist, Erastin, and reduced the formation of ROS and the accumulation of lipid peroxidation, and increased the number of mitochondria and enhanced the antioxidant activity, represented by GSH, of chondrocytes. GSH is the antioxidant level. Although some studies have shown that Osthole, an active ingredient in MQSD, can attenuate the phosphorylation levels of AMPK, Akt and mTOR in HCT116 and SW480 cells, inducing cellular ferroptosis and exerting anti‐tumour effects [46], these findings are specific to tumour cells. As previously mentioned, the regulation of the PI3K/Akt signalling axis exhibits a dual role in OA cartilage homeostasis. Therefore, these findings are not contradictory to our conclusions; rather, they underscore that MQSD can modulate Akt protein phosphorylation levels to exert its biological effects. However, the positive role of MQSD in chondrocytes warrants further investigation. To address this, we employed the PI3K/Akt activator Recilisib and inhibitor LY294002 for experimental validation. Our results demonstrated that IL‐1β and Recilisib‐treated chondrocytes exhibited significantly decreased viability, whereas chondrocyte viability was enhanced in the MQSD pretreated group. Additionally, qRT‐PCR and western blot analyses revealed the positive effects of MQSD on chondrocytes. Western blot results demonstrated that MQSD enhanced the expression of the ferroptosis regulator GPX4, consistent with immunohistochemical findings in mice. Similarly, MQSD exhibited a comparable anti‐ferroptosis effect to LY294002. In conclusion, MQSD may exert its positive anti‐OA effect by inhibiting chondrocyte ferroptosis through modulation of the PI3K/Akt axis.

In our pursuit to unravel the pivotal active ingredients responsible for the potent and targeted therapeutic effects of MQSD, we conducted molecular docking assays involving several identified active constituents and their interactions with specific target proteins. Through computer‐simulated docking analyses, we observed that oxypeucedan hydrate, Cimicifugoside and Notopterol exhibited robust binding activities with PIK3CD, AKT1 and GPX4 protein receptors. Consequently, we posit that MQSD's anti‐OA effects may be mediated through the concerted actions of these components.

Our study has provided initial insights into the interplay between MQSD, ferroptosis, the PI3K/Akt signalling axis and OA. Specifically, we have demonstrated that MQSD modulates chondrocyte ferroptosis to uphold metabolic homeostasis via the PI3K/Akt signalling axis, thereby impeding the progression of OA. While our preliminary findings underscore the efficacy of MQSD against OA, our in vivo experiments in mice have yet to delve deeply into the underlying mechanisms. Consequently, we remain vigilant about the efficacy of MQSD, prompting the imperative for future investigations. Moving forward, our research endeavours will focus on expanding sample sizes and conducting more comprehensive in vivo studies. Additionally, we aim to develop novel compounds based on MQSD for targeted therapy against OA, thereby enriching the arsenal of treatment options available for this complex condition. Such efforts hold promise in advancing therapeutic strategies for OA and warrant further exploration in future research endeavours.

## 5 Conclusions

In conclusion, our results indicate that MQSD significantly hinders OA advancement, likely through the regulation of ferroptosis in chondrocytes via the PI3K/Akt pathway. Critical molecular constituents such as oxypeucedanin hydrate, Cimicifugoside, and Notopterol are identified as fundamental to MQSD's effectiveness. These findings furnish initial understanding into the mechanistic underpinnings of MQSD's therapeutic benefits in OA, offering important directions for its clinical utilisation.

## Author Contributions

**Chen Zhuang:** data curation (equal), formal analysis (equal), methodology (equal), writing – original draft (equal). **Wen‐kai Li:** data curation (equal), formal analysis (equal), investigation (equal), methodology (equal). **Xiaojuan Geng:** data curation (equal), methodology (equal), software (equal). **Yu Pan:** project administration (equal), resources (equal), writing – review and editing (equal). **Lei Yang:** conceptualization (equal), project administration (equal), writing – review and editing (equal). **Chenxuan Hong:** writing – review and editing (equal). **Enli Li:** writing – review and editing (equal).

## Conflicts of Interest

The authors declare no conflicts of interest.

## Data Availability

Data are available on request from the authors.
